# Helping boys at-risk of criminal activity: qualitative results of a multi-component intervention

**DOI:** 10.1186/1471-2458-11-364

**Published:** 2011-05-23

**Authors:** Ellen L Lipman, Meghan Kenny, Erin Brennan, Susanne O'Grady, Leena Augimeri

**Affiliations:** 1 Department of Psychiatry and Behavioural Neurosciences, McMaster University, 1280 Main Street West, Hamilton, Ontario, L8S 4K1, Canada; 2 Offord Centre for Child Studies, Patterson 206, Chedoke Site, 566 Sanatorium Road, Hamilton, Ontario, L9C 1Y3, Canada; 3 Banyan Community Services, 681 Main Street East, Hamilton, Ontario, L8M 1K3, Canada; 4 Child Development Institute, Toronto, Ontario, Canada

**Keywords:** Crime prevention, Multi component intervention, Boys, Aggression, Pre-adolescent, Community-based intervention

## Abstract

**Background:**

This qualitative study examines parent and child experiences of participation in a multi-component community-based program aimed at reducing offending behaviour, and increasing social competence in boys 6 to 11 years old in Hamilton, Ontario, Canada. The program builds on the concept of crime prevention through social development, and includes structured groups for the identified boy, parents, and siblings.

**Methods:**

A sample of 35 families participating in the multi-component program took part in the qualitative study. Individual interviews with the boys, parents and siblings asked about changes in themselves, relationships with family and peers, and school after the group. Interviews were taped, transcribed and content analysis was used to code and interpret the data.

**Results:**

Parents reported improvement in parenting skills and attainment of more effective communication skills, particularly with their children. Parents also found the relationships they formed with other parents in the program and the advice that they gained to be beneficial. Boys who participated in the program also benefited, with both parents and boys reporting improvements in boys' anger management skills, social skills, impulse control, and ability to recognize potentially volatile situations. Both parents and boys described overall improvement in family relationships and school-related success.

**Conclusions:**

The qualitative data revealed that parents and boys participating in the multi-component program perceived improvements in a number of specific areas, including social competence of the boys. This has not been demonstrated as clearly in other evaluations of the program.

## Background

Aggressive and antisocial behaviours such as fighting and stealing occur more frequently among boys than girls, with an estimated prevalence ratio of 3-4 to 1 [1,2] Boys and girls with these behaviours frequently have a range of other difficulties, including other emotional and behavioural problems, and academic and social impairments. Problems may persist into adolescence and adulthood. For example, young boys with persistent problems with aggressive and antisocial behaviour followed longitudinally from age 7 to 32 had significantly worse physical and mental health outcomes than those with other patterns of these behaviours (e.g., aggressive and antisocial behaviours limited to childhood or arising in adolescence)[3]. Adult problems include difficulties with anxiety, depression, drug and alcohol dependence, attempted suicide, poorer self-rated general health and increased mental health service utilization. Poor educational achievement and school dropout, unemployment and involvement in criminal activities also occur [4,5].

The costs associated with these behaviours are high [6,7], and these children use more resources than children with other psychiatric disorders [8]. By adulthood, young children with antisocial behaviours may cost society ten times that of children without these behaviours [9]. Costs extend beyond the health and criminal justice systems, and include education and residential care sectors as well as the individual families of children with these behaviours and of those who may be victimized. The importance of early intervention and prevention strategies for these children and youth is clear[10].

Multiple risk factors for aggressive and antisocial behaviours have been well-documented [1,11]. These varied risk factors are distributed across the individual, family, peer and community domains. For example, biological risk factors such as neurochemicals (e.g., low central serotonin turnover), under arousal of the autonomic nervous system, low child intelligence, and learning difficulties (e.g., reading disorder) are considered to be individual risk factors. Hostile attribution to neutral social cues and early physical maturation (in girls) are other individual characteristics associated with increased problem behaviours. Family-related factors include familial aggregation of these behaviours, parenting behaviours such as poor parental supervision and inconsistent discipline, and pre- and peri-natal toxin exposure (e.g., maternal smoking). With respect to the peer and community domains, rejection by peers and association with antisocial peers, and family and neighbourhood socioeconomic disadvantage have been identified.

Bronfenbrenner emphasizes the important influence of the interactions of individuals in their various social contexts on human behaviour [12]. Similarly, while resilience was once considered to be a trait of an individual, it is now recognized that this trait is not solely a characteristic of the individual, but arises from characteristics and interactions in the environment [13]. For children and youth who exhibit aggressive behaviours, these environmental influences include interactions with individuals such as their parents and siblings, interactions with groups of people such as their peer group and school, and interactions with the larger community and society (e.g., poverty, unsafe neighbourhood), and overlap with the risk factor profile.

Approaches to early intervention and prevention that identify strengths and difficulties at each of these levels of interaction allow programs to be targeted to diminish the effects of identified risk factors and to amplify the influence of protective factors. Henggeler and colleagues assert that interventions focused on multiple ecological levels are the most tenable means of diminishing the population levels of serious juvenile delinquency [14]. Timing of the implementation of an intervention is critical, as there is a substantial amount of research indicating that the earlier an intervention is implemented, the more improved the child's behaviour is at home and at school, in turn resulting in an increased likelihood of preventing later criminal activity [15,16]. Such approaches are also consistent with identified principles of effective prevention which include the need for a comprehensive program (defined as multi-component and addressing critical domains including family, peers and community), a program that promotes positive relationships and that is appropriately timed (initiated early enough to impact development of problem behaviour) [17].

Multi-level intervention programs have demonstrated success in decreasing antisocial behaviours [18], though not consistently [19]. However many of these programs are aimed at adolescents, and there has been less research on multi-level interventions for younger children.

We report on an intervention program for high-risk children: boys from 6- to 11-years old who have been in trouble with the law, or are deemed to be at risk of same. The SNAP ^® ^(Stop Now and Plan) Under 12 Outreach Program (ORP) was developed in Toronto [20], based on the concept of crime prevention through social development and accounting for risk factors at various ecological levels that can lead to criminal activity [12]. The goals of the program are reducing offending behaviour and increasing social competence [21]. There are core SNAP ORP child and parent groups, and families can access additional activities/services offered as part of the program. Previous quantitative program evaluation has been positive [22-24]. For example, we demonstrated that boys receiving the SNAP ORP program improved significantly more than comparison boys (on the waiting list) on parent-rated "offending" behaviours (e.g., rule-breaking, aggression) but not on teacher ratings of the same outcomes[24]. There were no significant differences between SNAP ORP and waiting list boys on social competence ratings by parents or teachers, though all boys improved post-group.

Further understanding of how and why participants improve or what prevents improvement can be gained through qualitative methods. Increasing awareness of values, meanings and preferences of participating families enables identification of shared and unique processes experienced by participants, and opportunities for program improvement and modification. The objective of this paper is to present the results of qualitative interviews with participating boys, parents and siblings in an effort to further understand the impact of participation.

## Methods

The overall project was an embedded mixed methods study where both qualitative and quantitative methods were used to evaluate a multi-component program aimed at reducing offending behaviour, and increasing social competence in boys 6 to 11 years of age. The project included a replication of an earlier quantitative program evaluation done elsewhere [22] and a qualitative component. The program was run by Banyan Community Services in Hamilton, Ontario, Canada. Both the quantitative and qualitative components were completed by our independent academic third party [24]. For the qualitative study, principles of fundamental qualitative description [25] were used to guide the data collection and analysis processes. This type of qualitative approach is used to provide a comprehensive summary of facts and events, using the 'everyday' language of the participants, and is commonly used by researchers who require answers to questions about specific events or phenomena [25].

### Intervention Description: The Under 12 Outreach Program

This intervention has separate programs for boys and girls, as early experience with mixed gender groups suggested problems using this format[26,27]. We focus on the program aimed at boys.

The SNAP (Stop Now and Plan) groups are the core components of the SNAP ORP model, and are manualized cognitive behaviour therapy-based structured groups (Transformer Club for boys and SNAP Parent group for parents) that aim to teach self-control and problem-solving techniques to boys, while simultaneously teaching effective child management to parents.

The Transformer Club is offered to boys over a 12-week period, with a concurrent SNAP Parent Group. Each group includes seven boys between the ages of 6 and 11 years old, and is facilitated by two trained SNAP ORP Child Workers. Each Transformer Club meeting is divided into sections of unstructured play, discussion, modelling, coaching and behavioural rehearsal, structured play, and relaxation.

The SNAP Parents' Groups, facilitated by two trained ORP Family Workers, are run concurrently with the Transformer Club. In this group, parents learn the SNAP techniques that their children are learning, and parent management strategies to assist with managing their child's behaviour, through group discussion, modelling, role playing, homework assignments, and by viewing the videotapes of their child role playing applying SNAP.

A sibling group (Kidz Club) allows siblings aged 2 to 12 to participate in a group where they are introduced to the same SNAP concepts that are being covered in their brother's group, and are able to take part in pro-social recreational activities. This program component is co-facilitated by trained SNAP ORP staff and volunteers.

In addition to the core 12-week program SNAP ORP participants are offered access to several additional services including academic tutoring, clinical services, individual parent counselling, individual befriending (mentoring), school support/advocacy, community advocacy, and victim restitution.

### Participants

#### Quantitative

Details of the quantitative part of the study have been published elsewhere [24], and will be described briefly. The project was approved by the Research Ethics Board of Hamilton Health Sciences/Faculty of Health Sciences, McMaster University (REB # 05-032). Participants were recruited through community advertisement (newspaper, radio, local cable television) and suggestions to families by police, child welfare, school personnel and children's mental health services. To be eligible for the program, boys had to be 6-to-11 years of age, live in Hamilton, Ontario, Canada, and must have had police contact or be considered at risk of police contact based on elevated Child Behaviour Checklist (CBCL) or Teacher's Report Form (TRF) [28] (see below). Interested parents/boys meeting these eligibility criteria during a telephone interview were interviewed face-to-face within two weeks. Boys accepted in the program had police contact and/or elevated scores (clinical range, T score > 69) for "offending" behaviours (rule-breaking, aggressive behaviour and conduct) on the CBCL or TRF [28]. Boys with significant developmental delay or who were non-English speaking were excluded.

Quantitative data were collected between February 2002 and December 2005. Sessions began in February 2002, and ran three times a year (Winter, Spring and Fall). Each session consisted of three boys' groups (SNAP Children's Groups), parents' groups (SNAP Parent Group) and siblings' groups (Kidz Club). Each boys' group included seven boys (total n per session = 21, except session one = 17). Participants were scheduled for the next available session on a first-come, first-served basis.

During this phase of the study, pre-group data available from 223 boys and families, revealed that most families reported economic disadvantage (24.3% depended on government assistance as a form of income in the year prior to the study, 66.7% reported concerns about money), and lived in poor neighbourhoods (67.0%) [24,29]. Many families were headed by a lone parent (52.8%), usually a mother (76.9%). Half of the families reported current and/or past child welfare involvement (51.1%) [24].

#### Qualitative

For this qualitative assessment, the SNAP ORP program staff attempted to make initial contact with the 95 families who had enrolled in the SNAP ORP since our original evaluation (i.e., after December 2005) [24]. Program staff informed the families of the evaluation and that a research team member would be calling them to conduct an interview. Sixty (60) families were contacted by SNAP ORP staff and agreed to having a research team member call; others could not be reached or declined to be contacted. When the research team followed up with these 60 families, thirty five (35) families agreed to participate and were interviewed, including 42 parents (23 mother only, 5 father only, 7 families with both parents), 39 boys and 17 siblings. Reasons for non-participation in the qualitative study after contact by the research team included: declined to participate in an interview (8 families), could not be reached (10 families) and newly registered and had not yet participated in the core SNAP parent and child group programs (7 families).

### Data Collection

Interviews were conducted in the homes of each family by paired interviewers. Parents signed consent forms for their children to participate. The interviewers provided a brief explanation of the evaluation, why it was being done, and explained that the research team was independent from Banyan Community Services. Parents were also asked to consent to having the interviews audiotaped. Parents were interviewed first followed by their children. Children were asked if they preferred to have their parents present for their interview or if they were comfortable with talking with the interviewers alone while their parent(s) was in another room. All children completed interviews with their parents present.

**Figure 1:**
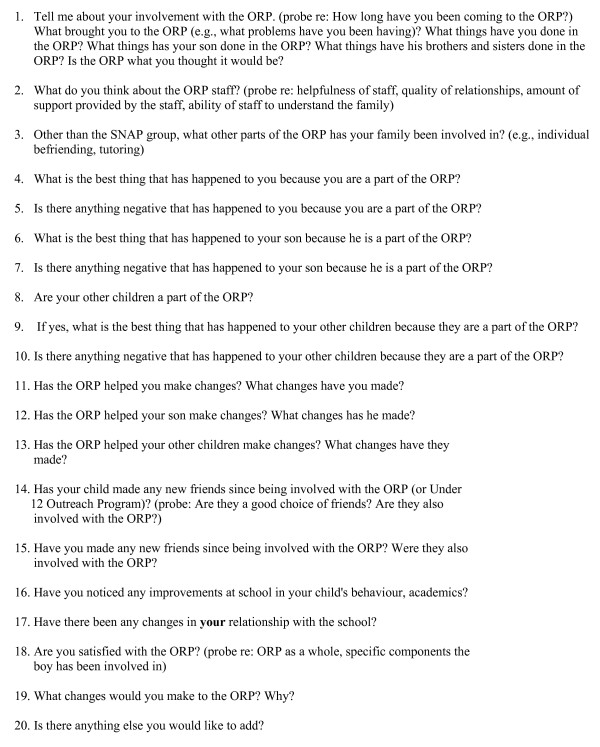
**Interview Guide for ORP Parents**.

**Figure 2:**
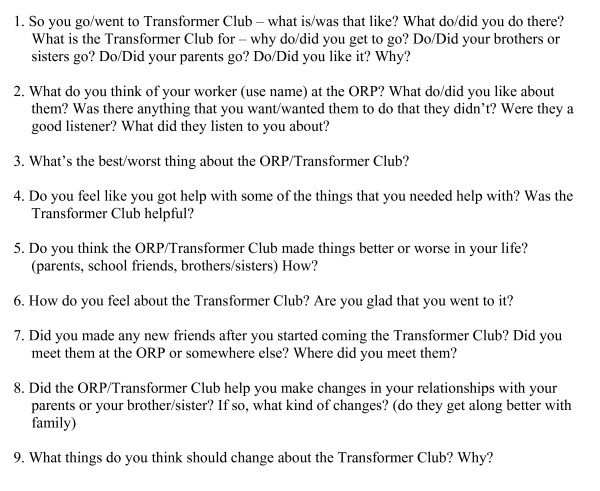
**Interview Guide for Children in Transformer Club**.

**Figure 3:**
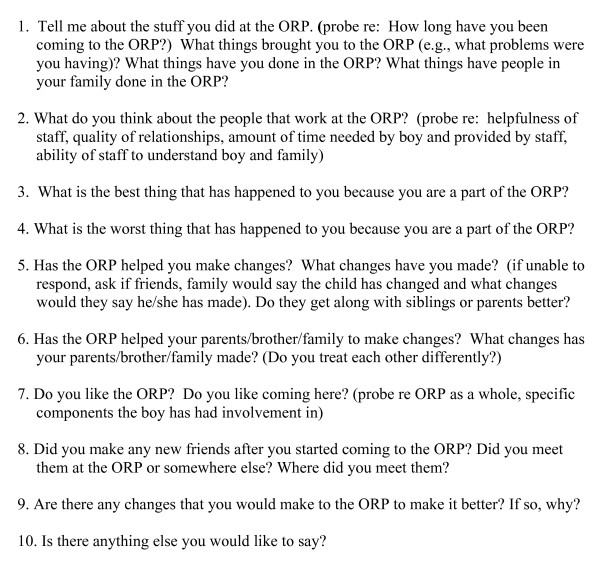
**Interview Guide for Siblings**.

In-depth, semi-structured interviews were completed using separate interview guides for parents and children, incorporating input from program staff members (see Figures 1, 2, 3). Parents were asked 20 questions ranging from general inquiries about their overall experience in the program, to more specific questions addressing any changes they may have witnessed in their children as a result of their involvement in the program. Children in the Transformer Club were asked nine questions ranging from what they liked/disliked about the program, to what changes, if any, the program helped them make. Siblings in the Kidz Club were asked ten questions about the impact of program participation on them, their brother and their family.

All of the interviews were audiotaped and transcribed verbatim. Interviews were done in pairs, with one person interviewing and the other taking notes. The head interviewer was female, held a master's degree (M.A.) and had extensive experience conducting qualitative interviews. The second interviewer was a female undergraduate psychology student. Each individual interview lasted 5-10 minutes for children and 30 - 60 minutes for parents.

### Analyses

In the qualitative analyses, a conventional content analysis approach was used in categorizing interview data. The main benefit of the conventional approach is allowing the researcher to draw information directly from the participants while refraining from applying any theoretical assumptions or predetermined inferences about the data [30]. While referring to their notes, interviewers reviewed all of the transcripts to ensure the accuracy of the transcription. Analysis of the data commenced with examining the interview transcripts and the interviewer notes. Preliminary codes emerging from the data were identified, while referring frequently to the interview guides and the evaluation questions to keep the context of the data in mind. Following this brief overview, phrases were highlighted in the transcripts and were viewed in light of the corresponding category, grouping all examples of a particular category together. Finally, all of the categories were listed, and examined in terms of more broad and overarching themes.

## Results

The main themes that emerged from the data regarding outcomes of participation in the SNAP ORP are reported below. They are divided into two sections, outcomes of participation for boys, and outcomes of participation for parents.

### Outcomes of Participation for Boys

#### Improved Anger Management Skills

One of the key outcomes associated with participation in the SNAP ORP was a newfound sense of control over their angry feelings. Boys who were once quick to react with anger and aggression are now applying the skills they have learned through their Transformer Club groups. Two parents described the change in their sons:

"[We] are seeing a difference in [our son] that he's calmer than he used to be. He's not quite as volatile and quite as explosive."

"[He'll] be just ready to explode and you'll just see him take the deep breath and calm down and start counting to himself and he'll think about it."

Several of the parents ascribed this improvement in impulse control to the boys learning to recognize the physical and situational warning signs for potentially volatile situations. As one parent explained,

"He can now probably assess his feelings a little better than just being angry and not knowing why. He can cognitively think a little differently and maybe deal with certain situations [that] he has dealt with [like] peer pressure."

#### Improved Relationships with Family Members

A result of the boys learning to manage their anger in a constructive manner has been an overall improvement in the relationships between parents and children. Some parents explained that when their son's behaviour got under control, they were able to focus on enjoying their time together. One boy described how the changes he has made have affected his family:

"...Since I'm not getting in trouble as much at school, we're all like doing stuff together and we're not yelling at each other as much as we used to because I used to get in trouble."

A parent also shared how the changes in her and her son have affected their relationship:

"...We both just seem a lot calmer with each other. We talk to each other a little more. We don't fight [and] we don't yell as much as we used to. Actually, I don't think we've yelled very much in the last while."

Another shared:

"My son and I have [grown] closer. That gap has been a little closed".

The families with whom we spoke also shared the changes they have noticed with sibling relationships as a result of learning the SNAP techniques. Some of the parents attributed this to siblings learning to better understand their brothers' needs. As one parent explained,

"I think [all of my kids] learned a better understanding of [my son] whether they realize it or not. I see them acting differently towards him and not just getting on his case because he does things wrong all of the time. They're starting to be more understanding...they've all learned a little bit more self-control [and] as I said, they're using SNAP."

One ORP boy stated:

"Like when [my brothers] bug me I don't like get mad."

#### School-Related Progress

Many of the parents that we interviewed commented on the noteworthy improvement in their sons' grades and overall behaviour at school. Parents were grateful for these improvements, as behavioural problems at school were a significant source of stress in their lives. Two parents talked about these changes:

"He had a school trip and...I went to volunteer and his teacher was like, oh my God, he's such a pleasure to be in my class, his grades have been getting better, he's been getting awards since we've been going to Banyan, like just 100% turnaround. Like when a teacher comes to me and says, my son is such a pleasure to be in her class, I'm like really? Not to insult my son or anything, but I've never heard that before, ever."

"There was literally times that day and day I was getting called from the principal and it was getting to the point where this was just too much...and there is a point where you can't do it anymore. But he has improved dramatically. It's been pretty good, he's been pretty good, he's been better."

Some parents noted an improvement in their relationship with the school. Fewer complaints from the school were coming to parents. As one parent said:

"I'm not [at the school] that often now. The only time I went there this year was to meet the teacher and that was it. That was the only time."

Some parents attributed the improvement to the presence of SNAP ORP workers providing school support and advocacy in the classroom and at meetings between parents and staff. Parents felt that because school staff members were aware of the effort being put forth by everyone involved with their child, they made more of an effort to be accommodating and understanding when their child was going through a particularly difficult time. As one mother shared,

"Because we are working with Banyan we find that because [the school] know[s] everything we are doing for [our son], even though he is getting suspended, yesterday he had a therapeutic leave because his sleeping has been bad. So they're lenient that way."

#### Improved Social Skills

It is not uncommon for children dealing with severe behaviour problems to experience difficulties maintaining friendships or forming new ones. An anticipated outcome for the boys participating in the SNAP ORP is for them to learn how to interact and socialize with their peers in a healthy manner. In speaking with parents and boys who had graduated from the SNAP ORP, it became apparent that boys were able to apply these new social skills and make new friends through the SNAP groups, as well as in other social contexts.

"I'm sharing more, smiling more [and] I'm not as aggressive...at recess I have a good time with my friends."

"[SNAP] helps me not to get in trouble as much and now all these kids want to play with me and it's better."

Parents also talked about the improvements they had noticed in their son's social lives:

"...Last year in school he had like no friends. They didn't want to play with him. This year he has quite a few friends in his class and in fact, we had one over here last night playing with him."

"So he's met friends [at group] and he has met more people since September again in general and part of it would be probably because of what he's learned with SNAP."

### Outcome of Participation for Parents

#### Improved Parenting Skills

One of the key outcomes that parents consistently commented on was their attainment of effective methods for which to communicate and effectively deal with their child's behaviour. Parents expressed a sense of relief and confidence knowing that they possessed the skills required to cope with any behavioural issues.

"I used to yell a lot. Now [I say], you know what? You want to be like that? Call me in five minutes and maybe we can talk about it. I've changed my approach in what we do. Like yelling just makes it worse. And I keep my voice just as calm as I possibly can and explain to him...if you don't do it this way, there is consequences to it one way or another you're going to understand that this is why we do things."

"Well, I think we're more positive with him instead of 'don't do this, and don't do that'. We're like okay; instead, we want this objective so how can we get there in a constructive way instead of a nagging [and] nattering way?"

A critical factor in parents honing their childrearing skills was having the opportunity to learn from other parents experiencing similar challenges with their children. Numerous parents expressed their appreciation for the chance to consort with others in their group, to seek advice and learn from their experiences.

"I found that meeting other parents it's great hearing their stories and hearing what they have done with their kids that has helped, and I've used a couple of their suggestions you know to see if it works with [our son]."

"Well we get a lot of support from other parents that are there. You know they have similar problems and that's why they're there. So we get similar ideas to different things you know. Like how to handle different situations. Some parents do it other ways. Some parents do it this way. You get different ideas from different people."

#### Establishing Relationships with Other SNAP ORP Parents

Many of the parents that we spoke with conveyed their gratefulness for the connections they had formed with other group participants. Being able to hear the stories of other parents trying to cope with similar challenges created a sense of decreased isolation and normalcy that these parents do not often get to experience.

"It's a really good support and we all kind of...when we are sitting in the room together we all, nobody, you get the feeling that nobody feels they are above anybody else. We are all coming from the same thing. We are all coping with the same problems; you know...just maybe some in more severity."

"I don't feel like I'm by myself now. Like hearing other people's stories I know that they're in the same boat."

Some parents formed close bonds with each other as a result of dealing with similar challenges while others vowed to stay in contact after completing the program. As two parents shared,

"We went to a meeting and one of the parents in my group had a serious thing happen. I was in tears and we consoled each other. It's like, you know what? That happened to me. It's like, wow. It's almost like so many years of searching and finding a solution with people who are the same. They're not different - they're just different problems. You're not alone."

"And some of us [in the group], when we are done, we'll be exchanging phone numbers because we know what's good for the kids because they're doing it together."

## Discussion

Disruptive behaviours in children such as aggression and antisocial behaviours are commonly associated with economic disadvantage [1,11,31]. Many families participating in this multi-component program aimed at reducing offending behaviour, and increasing social competence in boys were disadvantaged, as demonstrated by use of government assistance, worries about money, and area of residence. Many participating families were headed by lone mothers. Many lone mothers have low levels of education, employment and mood [32], though detailed questions on these characteristics were not gathered in this study.

Interview findings on a subgroup of families participating in the SNAP ORP program indicate that families perceive that the program is meeting one of its main objectives of increasing social competence in boys under the age of 12. Parents and children noted improvements in the children's anger management skills, which they attributed to their newly acquired SNAP strategies. Furthermore, both parents and boys reported the development of social skills and an increase in socially acceptable behaviour, which led to improved relationships with parents and siblings, and the formation of quality peer relationships. This latter outcome was particularly meaningful for parents, who shared the struggles that their children experienced trying to form or maintain friendships due to their inappropriate behaviour and/or lack of social skills prior to participating in the program.

Participants in the program also perceived that there were improvements in the children's academic performance and overall behaviour at school. Prior to entering the program many of the parents were overwhelmed by strained relationships with school staff members as a result of dealing with their child's behaviour problems. According to parents, the positive changes noted in their child's behaviour and academic performance alleviated some of the tension that existed between the home and school.

Parent involvement in the SNAP program is important. This was substantiated by the many parents who commented on how their newfound confidence and skill as a parent allowed them to effectively manage their child's behaviour. In addition to altering their behaviour management strategies, parents learned how to communicate with their children in a more effective and positive manner. By decreasing their negative interactions with their children, parents were able to enjoy their time with their children. The importance of parental involvement in programs for children and the association with better outcomes has been demonstrated by others [33].

Another positive outcome of participation discussed by parents was the decreased sense of isolation they experienced. Parents were able to connect with other families experiencing similar challenges and learn that they were not alone in their struggles. They were given an empowering opportunity to share their stories, support one another, and ultimately, learn from each other.

Previous quantitative evaluations of the SNAP ORP program in controlled studies have demonstrated positive changes in offending behaviours (e.g., [22,24]). Results on social competence outcomes, the other major goal of the program, are not reported in some studies [22,23], and have demonstrated non-significant improvements for boys [24] and significant improvements in girls [34,35]. In this qualitative assessment of program participation for boys, parents and boys reported that boys' social competence is improved, both within the family in terms of relationships with parents and siblings, as well as with peers and in the school setting. Parents also report feeling more competent, with improved parenting skills and reduction in the feeling that they are alone in experiencing these parenting challenges.

The program's attempt to address the various environments within which the child interacts may be an important factor in fostering a meaningful change in child behaviour and social interactions. Involvement of the entire family in the SNAP program (parent groups, focus child group, and sibling groups) may contribute to the improvements in family relationships. Involvement with the school is also beneficial. Involvement of younger children and the multiple environments targeted in the study (e.g., home, school) may decrease the likelihood of negative effects that have been demonstrated with bringing deviant children together in groups [36].

This paper describes qualitative research to better understand the impact of participation in a multi-component program aimed at reducing offending behaviour, and increasing social competence in boys on the boys, parents and siblings. This is, to our knowledge, the first time that mixed methods have been used to evaluate the SNAP Under 12 Outreach Program (ORP). A prominent theme emerging from the qualitative interviews with parents and boys participating in the program is that boys' social competence is improved. Our quantitative evaluation of boys in this program demonstrated non-significant improvements in social competence for boys [24]. If one considers the qualitative results as more strongly supporting improvements in social competence than the quantitative results, how can we reconcile these differences? This may be because each method of inquiry addresses a different question [37]. In quantitative studies the impact of ORP participation on offending behavior and social competence, based on CBCL and TRF measures, was examined. The CBCL social competency measures focus on engagement in community activities, social skills and school from the parent informant, and on behaviours associated with adaptive functioning ("works hard", emotional adjustment, learning and "how happy compared to other students of the same age") from the teacher. In this qualitative study we asked broader questions (e.g., What is the best thing that has happened to your son because he is a part of the ORP?). The theme of social competence emerged from the interview content. Outcomes not measured in the quantitative study were part of the qualitative study (e.g., making good choices of friends). Mixed methods approaches allow utilization of the strengths of each method while addressing methodologic limitations, offer complementary data and provide a fuller perspective of the impact of program participation on these families [37,38].

Limitations of this work should be noted. The 35 families who participated in the qualitative study were recruited after those described in the quantitative study and we only have the information collected in the interview about these families. There is no reason to suspect that these families differ sociodemographically from other families participating in the program previously; however, without actual assessments, we cannot rule out this bias. We conducted interviews with a sample of 35 families (42 parents, 39 boys and 17 siblings), but these families may not represent the views or experiences of all families who participated in the intervention. There was variable use of the additional broader program options by families and tracking of additional service use was incomplete so the extent to which participation in additional program components influences these results is not known. We had to rely on self-reports provided by participating parents and boys, without opportunity for external validation, and social desirability and parental expectancies may have influenced reporting.

## Conclusions

The findings of this qualitative study suggest that this multi-component program aimed at reducing offending behaviour and increasing social competence in boys is achieving the goal of increasing social competence. This has not been demonstrated as clearly in other evaluations of the program for boys [22,24]. Parents and children report learning strategies that they find useful and that they are applying in their homes, in their community, and in their schools. Participation in the program was perceived to contribute to improved child behaviour, improved parent management skills, and healthier family relationships. The convergence of the positive qualitative results reported in this paper with previous quantitative results is encouraging.

From a clinical standpoint, boys and families like those participating in our study frequently seek, or are encouraged to seek, assistance through clinical services and community agencies. Ideally, all community-based programs and other services should include an evaluation component in addition to service delivery. The ability to evaluate program impact of services provided using mixed methods offers the best opportunity for identifying effective programs and the most comprehensive understanding of program utility [39].

## List of Abbreviations

SNAP^®^ORP: Under 12 Outreach Program; SNAP^®^: (Stop Now and Plan)

## Competing interests

The authors declare that they have no competing interests.

## Authors' contributions

EL designed and conducted the study and drafted the manuscript. MK performed data collection, analysis and interpretation and assisted with manuscript review. EB assisted with data collection and manuscript review. SO and LA assisted with study conceptualization and manuscript review. All authors have read and approved the final manuscript.

## Pre-publication history

The pre-publication history for this paper can be accessed here:

http://www.biomedcentral.com/1471-2458/11/364/prepub
